# Evaluation of Protein Solubility, Lipid Oxidative Stability and Physical Properties of Hemp Seed‐Based Foods and By‐Products

**DOI:** 10.1002/fsn3.70954

**Published:** 2025-09-10

**Authors:** Ricardo Ramos‐Sanchez, Nicholas J. Hayward, Wendy R. Russell, Sylvia H. Duncan, Madalina Neacsu

**Affiliations:** ^1^ University of Aberdeen Rowett Institute, Foresterhill Campus Aberdeen UK

**Keywords:** lipid oxidation, particle size, physical properties, protein solubility, revalorization of hemp by‐products

## Abstract

Delivery of nutritious foods with a reduced carbon footprint is essential to deliver a circular food system. This paper represents a thorough analysis of protein solubility, lipid stability, and physical properties of four hemp by‐products and seven hemp seed foods to help evaluate their potential to replace less sustainable food ingredients like soya, wheat flour, and palm fat. The hemp seed‐hull flour and expellers had the highest values for loose bulk density (0.53 ± 0.01 g/cm^3^ and 0.49 ± 0.01 g/cm^3^) and tapped bulk density (0.68 ± 0.04 g/cm^3^ and 0.70 ± 0.02 g/cm^3^) across all the hemp samples, and were significantly higher than soya flour. The flowability of the hemp seed‐hull flour was better (passable) than that of wheat and the other hemp samples. The protein‐75‐product exhibited superior wettability properties (90% wettability at 0.83 ± 0.09 min). The cream solid residue (wet and dried) had the highest values for solubility (35.41% ± 10.21% and 35.55% ± 7.07%) and dispersibility (98.55% ± 1.28% and 99.20% ± 2.03%). The protein‐85‐product had the highest water‐holding capacity (2.10 ± 0.01 g/g powder). The hemp seed hearts sample had the highest protein solubility (13.74% ± 0.11%). The hemp fudge had the highest lipid oxidation stability (with 15.27 ± 1.65 h induction time) when compared to other hemp samples and also unrefined palm oil. The hemp seed ingredients and by‐products could be harnessed by the food industry to replace some palm oil, soya, and wheat‐based formulations in efforts to improve food sustainability.

## Graphical abstract

This paper represents a thorough analysis of protein solubility, lipid stability, and physical properties of four hemp seed by‐products and seven hemp seed foods to help evaluate their potential to replace less sustainable food ingredients like soya, wheat flour and palm fat. The present work brings strong evidence that support hemp seed ingredients and by‐products to be be harnessed by the food industry to replace ingredients like palm oil, soya and wheat‐based ingredients in food formulations in efforts to improve the food products sustainability.

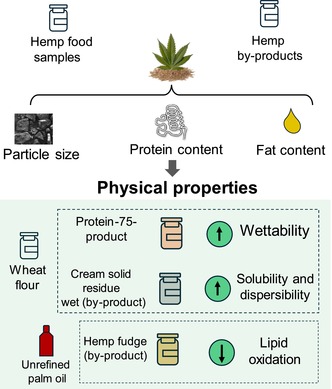

## 1 Introduction

The interest of consumers in having access to more natural, minimally processed low‐carbon footprint foods has led food companies to look for sustainable food ingredients that can be used to produce nutritious foods (Battacchi et al. 2020; Román et al. 2017). Agricultural hemp, in addition to its environmental credentials, represents a potential option to produce nutritious foods, as its seeds are rich sources of macro‐ and micronutrient compounds (Ramos‐Sanchez et al. 2025). Hemp seed contains, on average, 29.34% of dietary protein (Hwang et al. 2025), 33.02% of total fat (Rbah et al. 2024), and 24.18% of dietary fiber (Taaifi et al. 2021). In addition, hemp seeds are rich sources of micronutrients (Barčauskaitė et al. 2022), including potassium (900 mg/100 g), phosphorus (510 mg/100 mg), and magnesium (460 mg/100 mg). Processing hemp seeds results mainly in the production of hemp oil and hemp cake (hemp flour), which are commonly used for salad dressings (Crini et al. 2020) and baking goods (Lukin and Ksenia 2017; Sciacca et al. 2023). However, there are several by‐products that result from the hemp seed processing for food, such as hulls and expellers. The determination of nutrient composition and physical properties of hemp seed‐based samples is fundamental to determining their use in the food industry. Therefore, it is essential to assess how particle size, protein content, fat levels, and dietary fiber from hemp food powders influence production efficiency, consumer acceptance, and profitability in food industries (Siddiq et al. 2009). Bulk density, flowability, water‐holding capacity (WHC), oil‐holding capacity (OHC), wettability, solubility, and dispersibility are several types of physical properties that can be measured in distinct types of powder from hemp seed‐based samples. In addition, apart from physical properties, the stability of lipids from hemp seed‐based samples, particularly hemp oil or fat‐rich hemp samples, is crucial to be measured. Hemp oil has been reported to be a rich source of essential polyunsaturated fatty acids, including linoleic acid, gamma‐linolenic acid, and alpha‐linolenic acid (Crescente et al. 2018; Leizer et al. 2000; Oseyko et al. 2019). Nevertheless, food matrices containing unsaturated lipids are more likely to be oxidized when exposed to oxygen in light, air, and metal ions (Barden and Decker 2016; Kamal‐Eldin and Pokorny 2005; O'Connor and O'Brien 2006). This represents a health risk, as the deterioration of oils can lead to the development of rancid odors and unpleasant flavors and, as a result of the formation of toxic compounds, to the appearance of distinct types of health conditions, including cancer, cardiovascular and neurological diseases (Grootveld et al. 2020; Poyato et al. 2013). Moreover, the determination of protein solubility is essential, as it plays an important role in regulating physicochemical characteristics, processing, sensory qualities, shelf life, and nutritional composition of foods (Grossmann and McClements 2023).

The present work describes the physical properties of several hemp foods and main by‐products resulting from the hemp food industry, with the scope of evaluating their potential to be used as ingredients to substitute palm fat, soya, and wheat flour. Specifically, the impact of unsaturated fatty acids on the lipid stability of hemp cream, hemp fudge, hemp cake, and hemp oil has been assessed. Furthermore, the impact of particle size, fat and protein on bulk density, flowability, WHC, OHC, wettability, solubility, and dispersibility, along with protein solubility, was analyzed in powder form hemp samples. Explicitly, the hemp seed‐based foods analyzed were “protein fiber boost”, protein‐85‐product, protein‐75‐product, protein‐46‐product, hemp seed‐hull flour, hemp seed hearts, and whole hemp seeds. The hemp by‐products analyzed were expellers, hemp cake, and “cream solid residue” (wet and dried).

## 2 Materials and Methods

### 2.1 Chemicals and Materials

**FIGURE 1:**
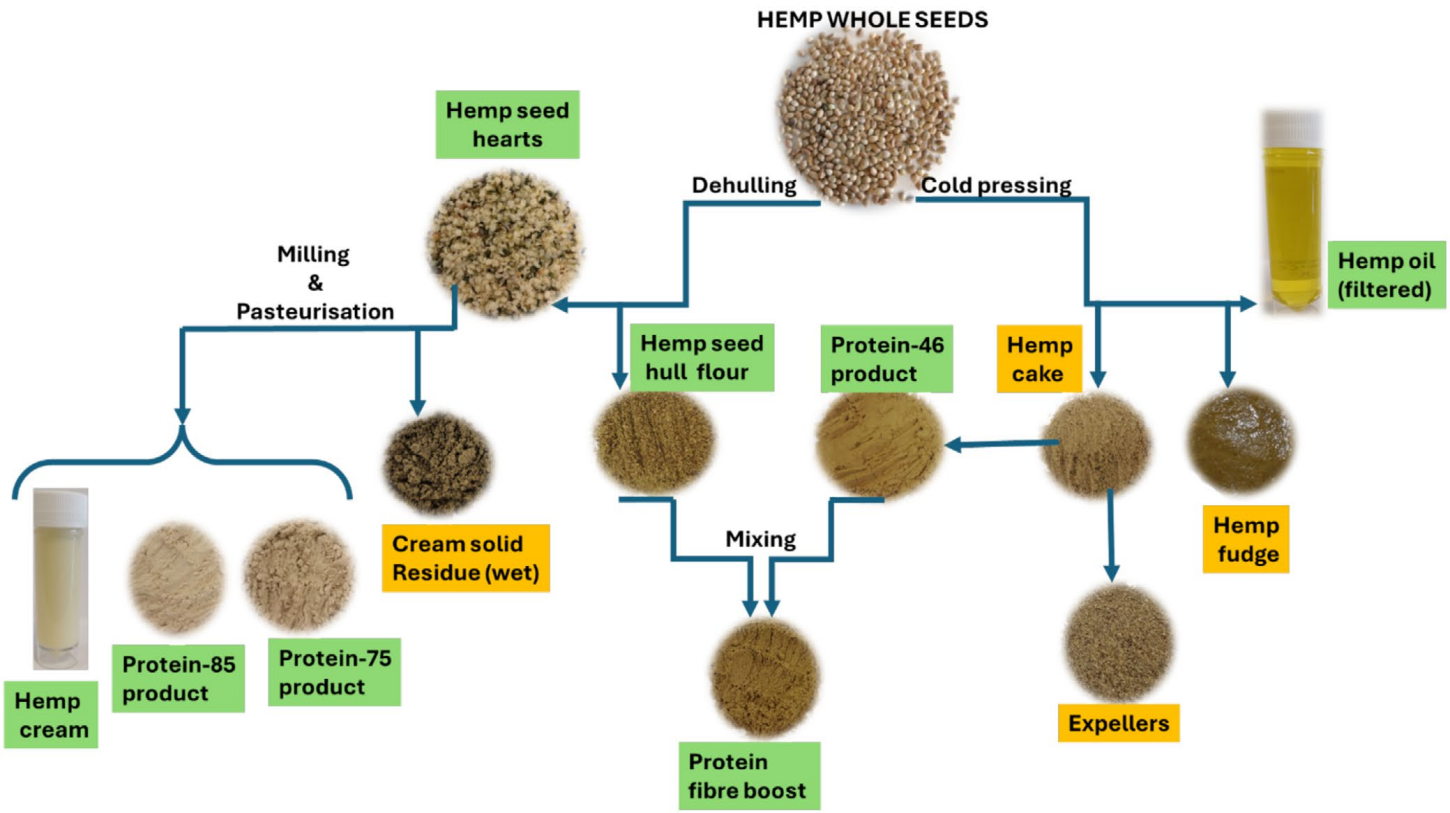
Schematic representation illustrating the production of different hemp seed‐based foods (green) and by‐products (orange) via milling and pasteurization and cold pressing.

All the hemp seed‐based samples were supplied by Braham & Murray Good Hemp (Barnstaple, UK), and the manufacturing processes used to obtain them have been previously published (Ramos‐Sanchez et al. 2025). Specifically, cold pressing and milling and pasteurization were used to produce different hemp seed‐based foods and by‐products (Figure 1). Unrefined palm oils, sunflower oil, wheat flour, and toasted soya flour were purchased from TESCO (Aberdeen, Scotland, UK). Organic hemp seed oil was purchased from Biona Organic (Kingston upon Thames, England, UK). Sodium dihydrogen phosphate monobasic dihydrate, disodium hydrogen phosphate, bovine serum albumin, and Bradford reagent used for protein solubility were purchased from Merck (Gillingham, England, UK). Electrodag 502, which was used to mount samples on stubs prior to the measurement of particle size, was purchased from Agar Scientific (Rotherham, England, UK).

### 2.2 Measurement of Particle Size Analysis of Hemp Seed‐Based Samples

The sample preparation for the determination of particle size was done using established methods published by other research groups (Varela et al. 2004). Freeze‐dried hemp seed‐based samples (*n* = 3) were mounted on stubs using Electrodag 502 (palladium Q15OT) and placed into a sputter coater (Quorum Q150T ES) for coating. Then, the samples were analyzed on the EVO MA10 scanning electron microscope (ZEISS; Oberkochen, Germany). Particle size in each of the images acquired was measured using the software ImageJ, with the results being reported in micrometers.

### 2.3 Determination of Loose and Tapped Bulk Densities of Hemp Seed‐Based Samples

The determination of loose and tapped bulk densities was done according to previous publications (Şahin‐Nadeem et al. 2013). The loose bulk density was determined by adding 2 g of hemp seed‐based sample (*n* = 3) into a 10 mL measuring cylinder (diameter 1.2 cm). The tapped bulk density was determined by gently transferring 2 g of hemp seed‐based sample (*n* = 3) into 10 mL measuring cylinders (diameter 1.2 cm), followed by tapping the measuring cylinders 120 times from a height of 15 cm on a rubber mat to promote particle compaction. The loose and tapped bulk densities were calculated by recording the volume occupied by the samples in the measuring cylinders.

(1) $$\mathrm{LBD}=\frac{\text{Powder weight}\;\left(g\right)}{\text{Loose powder volume}\;\left(\mathrm{mL}\right)}$$

(2) $$\mathrm{TBD}=\frac{\text{Powder weight}\;\left(g\right)}{\text{Volume after tapping}\;\left(\mathrm{mL}\right)}$$

### 2.4 Determination of Flowability of Hemp Seed‐Based Samples

The flowability was measured according to the Carr index and Hausner ratio indicators (Jinapong et al. 2008). These indicators are calculated from both loose and tapped bulk density results (*n* = 3).

(3) $$\mathrm{CI}=\frac{\text{Tapped density}-\text{loose bulk density}}{\text{Tapped density}}\times 100$$

(4) $$\mathrm{HR}=\frac{\text{Tapped density}}{\text{Loose bulk density}}\times 100$$

### 2.5 Determination of Wettability of Hemp Seed‐Based Samples

The analysis of wettability (*n* = 3) was done according to previous publications (Fitzpatrick et al. 2016). The evaluation of the wettability of powders that did not achieve complete wetness within 60 min and consequently did not sink was expressed as the time required to get 90% of wettability. This was achieved by assessing 90% of wettability at different time intervals, including 0–15 min, 15–30 min, 30–45 min, and 45–60 min. In this regard, 10 g of hemp seed‐based sample (*n* = 3) was sprinkled into 500 mL beakers containing 100 mL of distilled water (diameter 8 cm). Once most of the sample had sunk into the water, the time was recorded. For the samples that remained on the surface of the water after 60 min, some modifications were made. In this regard, 10 g of hemp seed‐based sample (*n* = 3) was sprinkled into 500 mL beakers containing 100 mL of distilled water. After 60 min, the powders that remained on the surface of the water were removed and taken to dryness in an oven at 100°C for 24 h. The samples were then taken out of the oven, weighed, and further factorized in their original water content to accurately calculate the quantity of powder that remained afloat. Then, the percentage of wettability was calculated.

### 2.6 Determination of Water Solubility of Hemp Seed‐Based Samples

The water solubility was measured according to previously published methods (Felix da Silva et al. 2018; Neacsu et al. 2022). Ten grams of hemp seed‐based sample (*n* = 3) was placed into 500 mL beakers (diameter 8 cm) containing 100 mL of distilled water. The mixtures were stirred continuously at 800 rpm for 7 min with an Ultra Turrax homogenizer (IKA T10, Staufen, Germany) and then allowed to stand for 30 s. Aliquots (20 mL) were transferred into tubes, and the samples underwent centrifugation at 1800 × g for 10 min. The supernatants obtained were placed into dry, pre‐weighed crucibles, and the samples were dried (100°C) overnight to constant weight. The crucibles were taken out and put into a desiccator to allow them to reach room temperature, and then the solubility was determined. Once the samples were cooled, they were reweighed, and the solubility was calculated.

(5) $$\%\text{Solubility}=\frac{\left(100+a\right)\%\mathrm{TS}}{a\left(100-b\right)/100}$$

where *a* is the amount of powder (g), *b* is the moisture content in the powder, and % TS is the percentage of dry matter in the supernatant.

### 2.7 Determination of Dispersibility of Hemp Seed‐Based Samples

The dispersibility was measured according to previously published methods (IDF 2014). An amount of 10 g of hemp seed‐based sample (*n* = 3) was put into 500 mL beakers (diameter 8 cm) containing 100 mL of distilled water. Mixtures were stirred continuously at 800 rpm for 7 min and then allowed to stand for 30 s. Then, the samples were aliquoted (20 mL) through a 60‐mesh sieve (around 210 μm). Filtrates obtained (8 g) were transferred into dry crucibles and taken to dryness overnight until constant weight at 100°C. Once the samples were cooled, they were weighed, and the dispersibility was calculated.

(6) $$\%\text{Dispersibility}=\frac{\left(100+a\right)\%\mathrm{TS}}{a\left(100-b\right)/100}$$

where *a* is the amount of powder (g), *b* is the moisture content in the powder, and % TS is the percentage of dry matter in the supernatant.

### 2.8 Determination of Oil Holding Capacity of Hemp Seed‐Based Samples

The OHC was determined according to previous publications (Robertson et al. 2000) with some modifications. Hemp seed‐based samples (1 g, *n* = 3) were weighed in pre‐weighed 50 mL plastic centrifuge tubes. Samples were dissolved in sunflower oil (20 mL) and mixed with a vortex at high speed (30 s). Samples were allowed to stand at room temperature for 30 min and then centrifuged at 1800 × g for 30 min. The supernatants from the samples were removed, and the mass (sample‐oil mixture) was recorded.

(7) $$\mathrm{OHC}=\frac{\mathrm{Wet}\;\text{powder weight}-\mathrm{dry}\;\text{powder weight}}{\mathrm{Dry}\;\text{powder weight}}$$

### 2.9 Determination of Water‐Holding Capacity of Hemp Seed‐Based Samples

The WHC was determined according to previous publications (Robertson et al. 2000) with some modifications. An amount of 1 g of hemp seed‐based sample (*n* = 3) was weighed out in pre‐weighed 50 mL plastic centrifuge tubes. Distilled water (10 mL) was added to the samples, mixed well for 30 s, and then allowed to stand at room temperature for 30 min. Then, the samples underwent centrifugation (1800 × g) for 30 min, and the supernatants were removed. Pellets were taken out of the tubes and then weighed out.

(8) $$\mathrm{WHC}=\frac{\mathrm{Wet}\;\text{powder weight}-\mathrm{dry}\;\text{powder weight}}{\mathrm{Dry}\;\text{powder weight}}$$

### 2.10 Assessment of Protein Solubility of Hemp Seed‐Based Samples

The protein solubility was determined according to previous publications (Morr et al. 1985). The bovine serum albumin standard solution was prepared in phosphate buffer (10 mM, pH 10) at different concentrations (between 0.1 mg/mL and 1.5 mg/mL). The hemp seed‐based samples (10 mg/mL, *n* = 3) were prepared in phosphate buffer (10 mM). The samples were stirred well and mixed on a roller mixer (SRT2, Salford Scientific, Manchester, UK) at room temperature for 30 min. Samples underwent centrifugation at 1800 × g for 10 min, and the supernatants were transferred into 1.5 mL tubes. The assay was done by adding 5 μL of hemp seed‐based sample and bovine serum albumin standard solutions onto a 96‐well plate in triplicate. Then, the Bradford reagent (250 μL) at room temperature was added to each well, and the plate was mixed on a shaker for 30 s. The samples were then incubated at room temperature for 20 min, and after that, the absorbance was read at 595 nm (Infinite M Nano, Tecan, Grödig, Austria).

### 2.11 Measurement of Lipid Oxidation in Hemp Fudge, Hemp Cake, Hemp Oil, and Hemp Cream

Lipid oxidation was determined according to previous publications (Ghosh et al. 2019). A 743 Rancimat instrument (Metrohm; Herisau, Switzerland) was used for the analysis of lipid oxidation. The Rancimat was set at 120°C, with a gas flow of 20 L/h and a start delay of 5 min. Measuring vessels containing 60 mL of milli‐Q water were collocated in the instrument and securely sealed with their respective lids, ensuring that metal pins were also inserted. Then, 3 g of hemp seed‐based sample (*n* = 3) was put into glass reaction tubes fitted with glass tubes to allow the entry of air into the samples. Glass reaction tubes were attached to the measuring vessels using silicone tubes. Once the temperature reached 120°C, the glass reaction tubes were then connected to the Rancimat instrument, and the samples were run for up to 24 h to measure their induction time.

### 2.12 Statistical Analysis

All the samples were analyzed in triplicate, and the results are reported as mean ± standard deviation. Significant differences in the physical properties, protein solubility, and oxidative stability were assessed by a one‐way analysis of variance (ANOVA, (*p* < 0.05)) and Tukey's test. Significant differences in particle sizes were assessed by the Kruskal‐Wallis test (*p* < 0.05). Minitab Statistical Software (×64)—21.1.1.0, Microsoft Excel for Office 365 (Microsoft Corporation, Redmond, Washington, DC, USA), and GraphPad Prism (version 10.2.3) were used for statistical analysis.

## 3 Results and Discussion

### 3.1 Particle Properties

The shape of all the hemp seed‐based samples can be described as rather anisotropic (Figure S1). Particle analysis of the hemp seed‐hull flour, hemp seed hearts, expellers, protein fiber boost, and seeds revealed a well‐dispersed morphology with limited agglomeration and well‐defined edges, suggesting low cohesive character. The particles of the protein‐75‐product, protein‐85‐product, protein‐46‐product, and hemp cake exhibited a certain degree of agglomeration, with particles adhering to one another, likely due to their smaller particle sizes (Table 1). Additionally, the particles of the cream solid residue (wet and dried) exhibited a more pronounced agglomeration, which could be attributed to the high fat content present in these samples (Table 1). The content of fat, particle size, and protein are critical factors determining the particle characteristics of the hemp seed‐based samples. As demonstrated in the following sections, these particle properties are closely associated with the physical properties of the hemp powders.

**TABLE 1:** Wettability (%) of the hemp seed‐based samples, toasted soya flour and wheat flour expressed as means (±SD, *n* = 3) and the measurements that can modify the wettability in powders: total fat (%), protein (%) and particle size (um) as means (±SD, *n* = 3).

Sample	Time (min) for 90% wettability	Wettability (%) after 60 min	Measurements that can modify wettability		
			Total fat (%)^§^	Protein (%)^§^	Particle size (μm)
Protein fiber boost	52.10 ± 1.01^a^	99.72 ± 0.14^d^	16.60 ± 0.19^f^	38.10 ± 0.13^d^	160.92 ± 61.96^ab^
Protein‐75‐product	0.83 ± 0.09^e^	99.96 ± 0.00^ab^	10.90 ± 0.18^h^	74.50 ± 0.29^b^	69.32 ± 23.84^cd^
Protein‐85‐product	1.45 ± 0.02^e^	99.98 ± 0.00^a^	3.22 ± 0.18^j^	93.10 ± 0.18^a^	43.34 ± 34.78^d^
Protein‐46‐product	51.66 ± 1.52^a^	99.88 ± 0.03^abc^	13.40 ± 0.35^g^	55.60 ± 0.14^c^	81.68 ± 42.89^bd^
Hemp seed‐hull flour	28.70 ± 1.53^c^	99.72 ± 0.03^d^	8.59 ± 0.138^i^	13.50 ± 0.10^i^	274.71 ± 176.47^a^
Hemp seed hearts	5.12 ± 0.06^e^	99.90 ± 0.01^abc^	45.50 ± 0.60^c^	35.80 ± 0.42^e^	385.78 ± 240.78^a^
Expellers	2.70 ± 0.35^e^	99.77 ± 0.00^cd^	10.10 ± 0.59^hi^	25.10 ± 0.19^h^	325.30 ± 180.53^a^
Seeds	1.08 ± 0.10^e^	99.84 ± 0.02^bcd^	30.90 ± 1.66^e^	26.60 ± 0.19^g^	314.80 ± 234.26^a^
Hemp cake	1.39 ± 0.05^e^	99.81 ± 0.01^cd^	10.70 ± 0.18^h^	34.40 ± 0.19^f^	79.78 ± 40.65^cd^
Cream solid residue (wet)	5.00 ± 0.50^e^	99.97 ± 0.00^a^	41.30 ± 0.39^d^	35.60 ± 0.29^e^	171.11 ± 76.18^a^
Cream solid residue (dried)	3.30 ± 0.26^e^	99.95 ± 0.00^ab^	42.20 ± 0.23^d^	35.90 ± 0.04^e^	199.90 ± 97.93^abc^
Toasted soya flour	18.66 ± 3.21^d^	100.00 (at 38 min)^†^	9.00^‡^	53.60^‡^	52.27 ± 40.96^d^
Wheat flour	41.33 ± 7.37^b^	99.88 ± 0.00^abc^	1.50^‡^	9.70^‡^	42.42 ± 18.67^d^

*Note:* Data within the same column with different letters are significantly different (*p* < 0.05).

^§^ The fat and protein content among all the hemp seed‐based samples has been previously published (Ramos‐Sanchez et al. 2025).

^†^ The toasted soya flour met 100% wettability at 38 min.

^‡^ Where percentages of total fat and protein in toasted soya flour and wheat flour were obtained from the nutritional information labels of their commercial brands, therefore ±SD was not included.

### 3.2 Loose Bulk Density and Tapped Bulk Density

**FIGURE 2:**
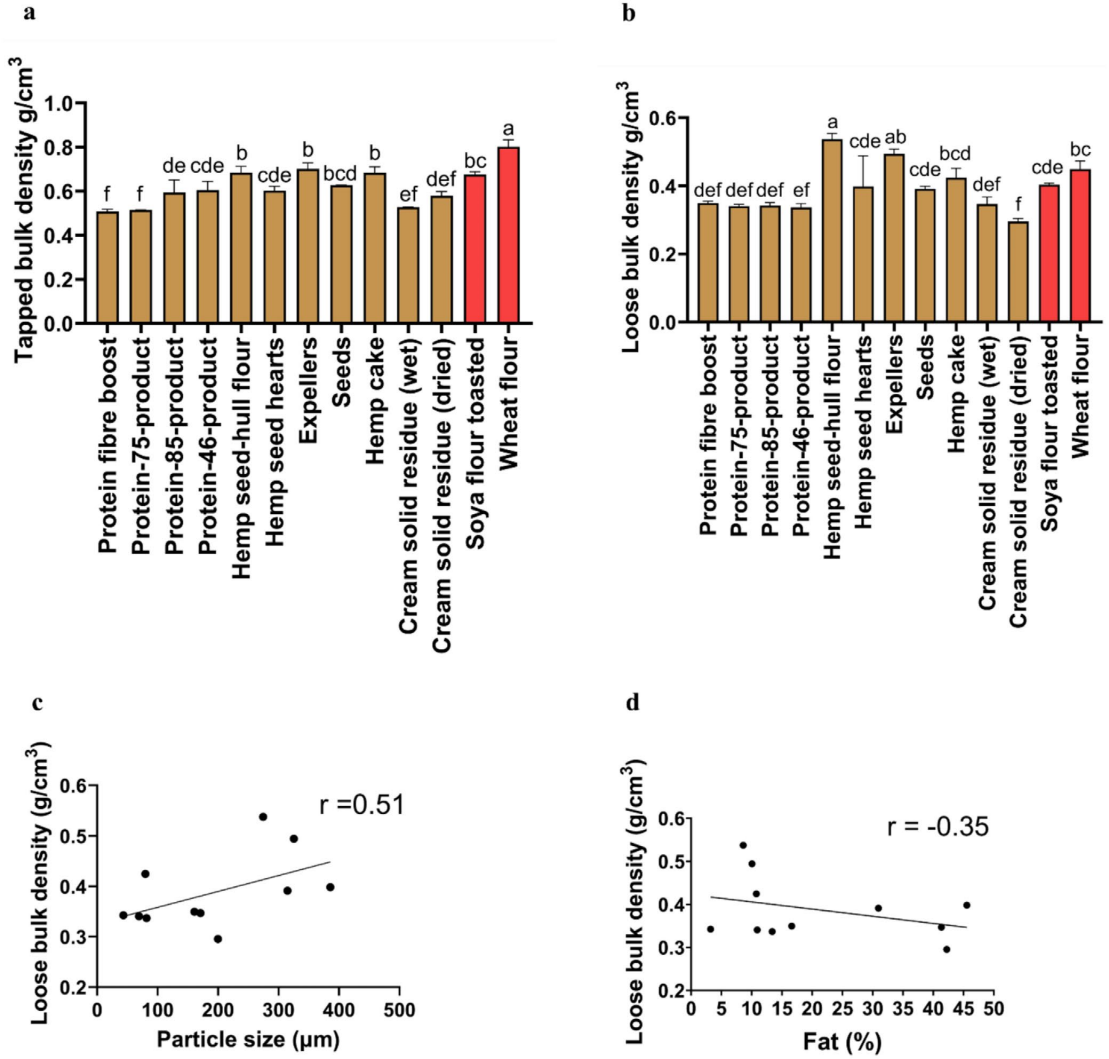
Bulk density of hemp seed‐based samples, toasted soya flour, and wheat flour expressed as means (±SD, *n* = 3). (a) Tapped bulk density (g/cm^3^); (b) loose bulk density (g/cm^3^); (c) Pearson correlation coefficient between loose bulk density (g/cm^3^) and particle size (μm); (d) Pearson correlation coefficient between loose bulk density (g/cm^3^) and fat (%). Each point in c and d represents a different hemp seed‐based sample expressed as means (*n* = 3). Distinct letters above the bars (a–f) indicate statistically significant differences (*p* < 0.001). The fat content among all the hemp seed‐based samples (d) has been previously published (Ramos‐Sanchez et al. 2025).

The tapped bulk density measurements were significantly higher (*p* < 0.001) in hemp seed‐hull flour (0.68 ± 0.04 g/cm^3^), expellers (0.70 ± 0.02 g/cm^3^), and hemp cake (0.68 ± 0.02 g/cm^3^) compared to the rest of the other hemp seed‐based samples (Figure 2a). The tapped bulk density in the wheat flour (0.80 ± 0.03 g/cm^3^) was significantly higher (*p* < 0.001) compared to the tapped bulk density of the hemp seed‐based samples (Figure 2a). Moreover, the tapped bulk density of six out of 11 of the hemp seed‐based samples was similar to toasted soya flour. Furthermore, the tapped bulk density results obtained among all the hemp seed‐based samples and wheat flour were similar to those of a study on hemp seed flour previously published (0.71 g/cm^3^) (Merlino et al. 2022).

The tapped bulk density of the hemp seed‐based samples (Figure 2a) was similar to previously reported values on plant‐based protein for oat flour (Hamdani et al. 2014), hulled barley flour (Hamdani et al. 2014), dehulled barley flour (Hamdani et al. 2014), and sesame protein isolate (Ghorbani et al. 2025), which ranged from 0.47 g/cm^3^ to 0.72 g/cm^3^, respectively.

The loose bulk density (Figure 2b) was found to be significantly higher in hemp seed‐hull flour (0.53 ± 0.01 g/cm^3^) and expellers (0.49 ± 0.01 g/cm^3^) in comparison with the rest of the other samples (*p* < 0.001). Moreover, the loose bulk density in eight out of 11 of the hemp seed‐based samples was similar to toasted soya flour, while in four out of 11 of the hemp seed‐based samples, it was similar to wheat flour. The bulk density of food powders might vary based on the particle size and amount of fat they contain. Generally, as the particle size increases, the arrangement of particles in a material becomes better; therefore, bulk density increases (Capece et al. 2014). In this regard, the particle size and fat content results (Table 1) indicated a positive Pearson correlation coefficient (*r* = 0.51) for loose bulk density among all the hemp seed‐based samples (Figure 2c), which suggests particle size may be associated with an increase in loose bulk density. On the other hand, the loose bulk density of the hemp seed‐based samples may have also changed due to the presence of fat (Table 1), as a negative correlation (*r* = −0.35) was observed between loose bulk density and fat content (Figure 2d).

### 3.3 Flowability

**FIGURE 3:**
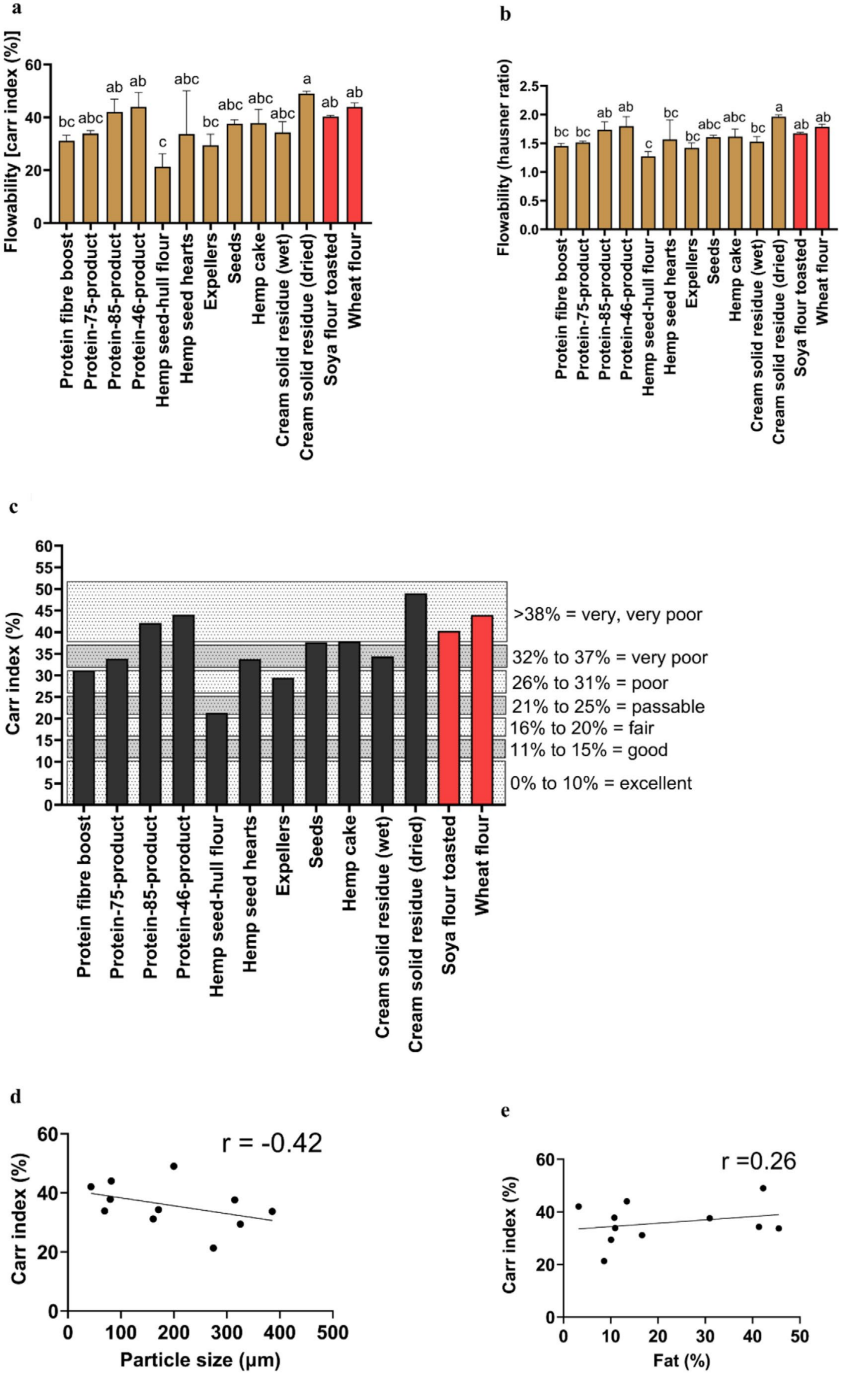
Flowability of hemp seed‐based samples, toasted soya flour, and wheat flour expressed as means (±SD, *n* = 3): (a) Flowability (Carr index in %); (b) flowability (Hausner ratio); (c) flow character based on the Carr index (%); (d) Pearson correlation coefficient between Carr index (%) and particle size (μm); (e) Pearson correlation coefficient between Carr index (%) and fat (%). Each point in (d) and (e) represents a different hemp seed‐based sample expressed as means (*n* = 3). Distinct letters above the bars (a–c) indicate statistically significant differences (*p* < 0.05). The fat content among all the hemp seed‐based samples (e) has been previously published (Ramos‐Sanchez et al. 2025).

The hemp seed‐hull flour had the lowest Carr index and Hausner ratio indicators among all the samples (Figure 3a,b). These results indicated that the hemp seed‐hull flour had the best flowability (passable with a Carr index of 21.34%), as shown in Figure 3c. Generally, as the particle size increases, powders change from a cohesive stage to a more free‐flowing stage (Liu et al. 2008). This in turn signifies that powders having higher particle sizes will have lower Carr index values, thereby exhibiting superior flow characteristics (Lebrun et al. 2012). In this regard, the Pearson correlation coefficient in all the hemp seed‐based samples indicated that as the particle size increased (Figure 3d), the Carr index parameter decreased (negative correlation, *r* = −0.42), denoting an increase in the flowability of all the hemp seed‐based samples. Food samples with a high content of fat are associated with poor flowability (Kim et al. 2005). In this regard, the Pearson correlation coefficient between the Carr index and fat among all the hemp seed‐based samples (Figure 3e) demonstrated a weak positive correlation (*r* = 0.26). For example, the flowability of the expellers, according to its fat content of 10.06% (Table 1) and its Carr index value (29.44%), was shown to be poor (Figure 3a,c). Moreover, the cream solid residue (dried), whose fat content (42.20%) was higher than that of expellers (Table 1), was found to have the lowest flowability among all the samples analyzed (very, very poor, Carr index > 38%) (Figure 3c). On the other hand, the Carr index values of all the hemp seed‐based samples (Figure 3a) indicated that their flowability was lower than that of a published study on hemp seed flour (Dedebas and Cebi 2024), which reported a lower value for the Carr index parameter (7.14%) (Dedebas and Cebi 2024).

The hemp seed‐based samples demonstrated to have similar flowability characteristics to those reported previously on different plant‐based proteins commonly used for food consumption (Hamdani et al. 2014), including oat flour (poor, Hausner ratio = 1.38), hulled barley (very poor; Hausner ratio = 1.58), and dehulled barley flour (very poor; Hausner ratio = 1.52). Nevertheless, when compared to rice flour (good, Hausner ratio = 1.18) (Düsenberg et al. 2023), the hemp seed‐based samples showed reduced flowability, which is probably attributed to the lower fat content of rice flour (0.9%).

### 3.4 Wettability

The hemp seed‐based samples met at least 99% of wettability after 60 min (Table 1). The time to meet 90% of wettability of the toasted soya flour was significantly lower compared to that of the protein fiber boost, protein‐46‐product, and hemp seed‐hull flour but significantly higher compared to the rest of the other hemp seed‐based samples (*p* < 0.05). The time to meet 90% of the wettability of the wheat flour was significantly lower (*p* < 0.05) compared to that of the protein fiber boost and protein‐46‐product but significantly higher compared to the rest of the other hemp seed‐based samples (Table 1).

The time to meet 90% wettability was similar in different types of hemp seed‐based samples, including protein‐75‐product (0.83 ± 0.09 min), protein‐85‐product (1.45 ± 0.02 min), hemp seed hearts (5.12 ± 0.06 min), expellers (2.70 ± 0.35 min), seeds (1.08 ± 0.10 min), hemp cake (1.39 ± 0.05 min), cream solid residue (wet and dried) with 5.00 ± 0.50 min, and with 3.30 ± 0.26 min, respectively. The time to meet 90% wettability of the protein fiber boost (52.10 ± 1.01 min) and the protein‐46‐product (51.66 ± 1.52 min) was significantly higher (*p* < 0.05) compared to that of the rest of the hemp seed‐based foods and by‐products (Table 1). The wettability of powders with larger particle sizes tends to increase as water can penetrate easily between the void spaces between the particles of powders (Fitzpatrick and Cuthbert 2004; Jeantet et al. 2010; Richard et al. 2013; Schober and Fitzpatrick 2005). Interestingly, there was no observed reduction in the time to meet 90% of wettability for the protein fiber boost (Table 1), even though its particle size (160.92 ± 61.96 μm) was much larger than that of the protein‐46‐product (81.68 ± 42.89 μm). Besides, even though the amount of protein in the protein‐46‐product (55.60%) was found to be much higher (*p* < 0.05) than that of the protein fiber boost (38.10%), an increase in the time to meet 90% wettability for the protein‐46‐product was not observed (Table 1). These results suggest that possibly other factors (Fitzpatrick et al. 2016), including the contact angle (measurement of hydrophobic surface effects) or the apparent density, may be related to the differences observed between the wettability of these two hemp samples.

The content of fat is another factor that can modify the wettability of different powders. In this regard, as the content of fat increases, the hydrophobicity of the determined samples will increase (Fitzpatrick and Cuthbert 2004; Jeantet et al. 2010; Richard et al. 2013; Schober and Fitzpatrick 2005). In this study, the fat content between the hemp seed‐hull flour and the seed powder was found in similar amounts (Table 1), and also the amounts of protein were not too much different between them (13.50% and 26.60%), so the increase in wettability of the seed powder (Table 1) may be attributed to its particle size (314.80 ± 234.26 μm), which was higher compared to the particle size of the hemp seed‐hull flour (274.71 ± 176.47 μm). The time to meet 90% wettability in the by‐products of cream solid residue (wet) and cream solid residue (dried) was found to be similar (5.00 ± 0.50 min and 3.30 ± 0.26 min). The results between these two by‐products are not surprising, as their content of fat (41.30% and 42.20%), amount of protein (35.60% and 35.90%), and particle size (171.11 ± 76.18 and 199.90 ± 97.93) were similar (Table 1).

Previous research has examined the wettability of protein‐rich plant samples, demonstrating that both sesame meal and sesame protein isolate exhibited superior wettability properties (Ghorbani et al. 2025) compared to hemp seed‐based samples (Table 1). Based on particle size measurements, the sesame protein isolate—with a particle size from 0.2 μm to 4.4 μm—would be expected to display lower wettability than the hemp seed‐based samples, which exhibited larger particle sizes (Table 1). The enhanced wettability found in both the sesame meals and sesame protein isolate (Ghorbani et al. 2025) may be attributed to differences in the methodology used. Specifically, both sesame meal and sesame protein isolate (Ghorbani et al. 2025), in comparison with the hemp seed‐based samples (Table 1), were stirred using a magnetic stirrer, which possibly caused faster sedimentation of the samples, resulting in improved wettability (wettability considered as good as all the sample material sunk after 30 min).

### 3.5 Solubility and Dispersibility

**FIGURE 4:**
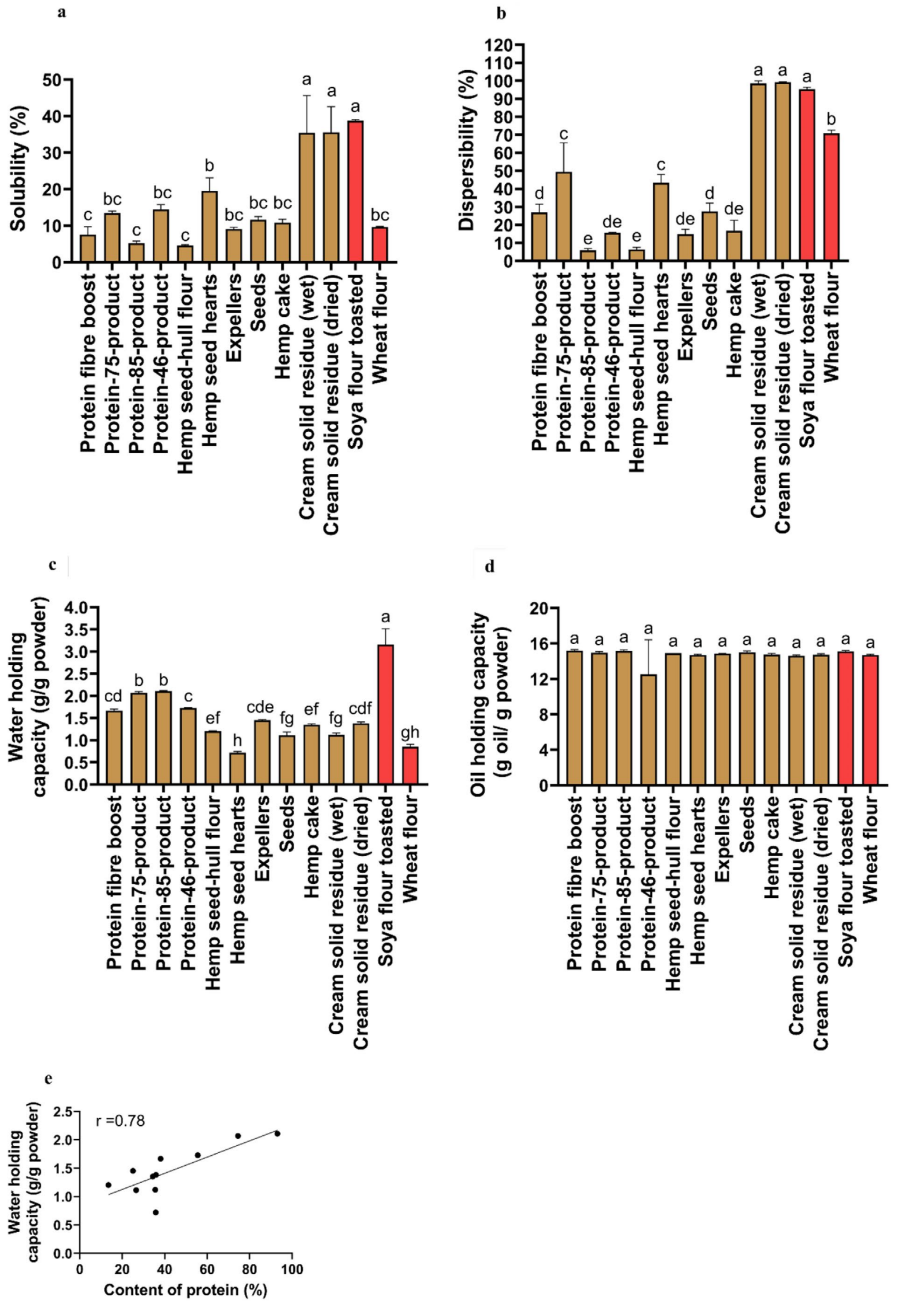
Physical properties of the hemp seed‐based samples, toasted soya flour and wheat flour related to hydration and expressed as means (±SD, *n* = 3): (a) solubility (%); (b) dispersibility (%); (c) water holding capacity (g/g powder); (d) oil holding capacity (g oil/g powder); (e) Pearson correlation coefficient between content of protein (%) and water holding capacity (g/g powder). Each point in (e) represents a different hemp seed‐based sample expressed as means (*n* = 3). Distinct letters above the bars (a–h) indicate statistically significant differences (*p* < 0.05). The protein content among all the hemp seed‐based samples (e) have been previously published (Ramos‐Sanchez et al. 2025).

The solubility values (35.41% ± 10.21% and 35.55% ± 7.07%) of the cream solid residue (wet and dried) were significantly higher than those of the rest of the other hemp seed‐based samples and wheat flour (Figure 4a). The solubility values of the wheat flour were similar in nine out of 11 of the hemp seed‐based samples. The solubility of the hemp seed hearts (19.51% ± 3.59%) was significantly higher (*p* < 0.05) than that of the protein fiber boost (7.55% ± 2.15%), protein‐85‐product (5.23% ± 0.54%), and hemp seed‐hull flour (4.54% ± 0.27%).

The highest percent of dispersibility was obtained by the cream solid residue (wet) with 98.55% ± 1.29%, the cream solid residue (dried) with 99.21% ± 2.04%, and the toasted soya flour with 95.34% ± 1.05%, thereby representing the samples with the best hydration in water (Figure 4b). Following this, the second‐highest percentage of dispersibility was obtained by the protein‐75‐product (49.51% ± 16.05%) and the hemp seed hearts (43.41% ± 4.48%). The lowest percent of dispersibility was obtained by the protein‐85‐product (5.84% ± 1.10%) and the hemp seed‐hull flour (6.33% ± 1.24%). Moreover, except for cream solid residue (wet and dried), the percent of dispersibility in the wheat flour (70.83% ± 1.74%) was significantly higher (*p* < 0.05) than that of the rest of the hemp seed‐based samples (Figure 4b).

### 3.6 Water‐Holding Capacity and Oil Holding Capacity

The WHC of the toasted soya flour was significantly higher compared to that of the hemp seed‐based samples and wheat flour (*p* < 0.05). The WHC (Figure 4c) of the protein‐75‐product (2.07 ± 0.03 g/g powder) and protein‐85‐product (2.11 ± 0.02 g/g powder) was significantly higher (*p* < 0.05) than that of the rest of the hemp seed‐based samples and wheat flour. On the other hand, the higher the content of protein, the better the WHC (Damodaran 2017; Khalid and Elharadallou 2013). In this regard, the Pearson correlation coefficient between the WHC and the protein content among all the hemp seed‐based samples (Figure 4e) showed a strong positive correlation (*r* = 0.78), thereby suggesting an increase in the WHC as the protein content increased. Moreover, the WHC found in the protein‐75‐product and in the protein‐85‐product was similar to other research on hemp flour (2.27 g/g and 2.25 g/g), which was obtained through organic and conventional agricultural methods (Absi et al. 2023).

Comparing our results for WHC (Figure 4c) with those reported in a previous study (Jacobs et al. 2015), a trend toward an increase in WHC was observed in wheat bran (Jacobs et al. 2015) as particle size increased (from 500 μm to 1500 μm), with values ranging from 4.2 g/g powder to 5.5 g/g powder, respectively. Nevertheless, in our study (Figure 4c), no linear trend was observed in the increase of WHC as particle size rose (Table 1). This suggests that different factors, such as protein content, were possibly associated with WHC behavior.

All the samples evaluated had similar values for OHC. Specifically, the protein fiber boost (15.20 ± 0.10 g oil/g powder) had the highest values for OHC compared to the rest of the other samples analyzed (Figure 4d). Moreover, the OHC of all the hemp seed‐based samples (Figure 4d) was substantially higher in comparison to the OHC reported in a published work on hemp flour (1.19 g/g and 1.26 g/g), whose sample was obtained through organic and conventional agricultural methods (Absi et al. 2023).

### 3.7 Protein Solubility

The protein solubility was significantly higher (*p* < 0.05) for the hemp seed hearts (13.74% ± 0.11%) followed by the protein fiber boost (11.12% ± 0.61%), compared to the other hemp‐based samples. The protein solubility of the expellers (9.94% ± 0.52%) was similar to that of hemp cake and hemp fudge but significantly higher (*p* < 0.05) than that of the cream solid residue (wet) and the cream solid residue (dried).

**FIGURE 5:**
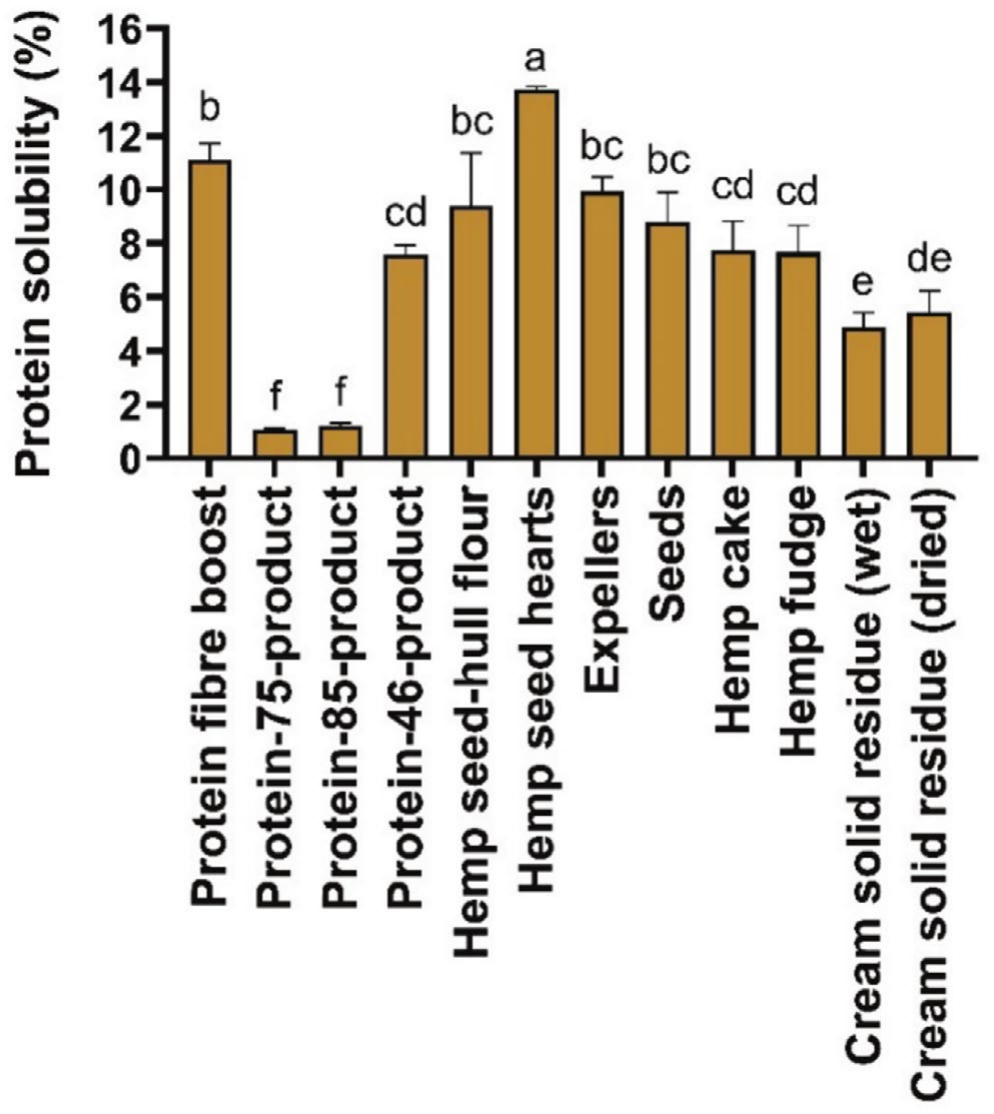
Protein solubility (%) of hemp seed‐based samples expressed as means (±SD, *n* = 3). Distinct letters above the bars (a–f) indicate statistically significant differences (*p* < 0.05).

The pH for which the protein solubility was tested for all the hemp seed‐based foods and by‐products was pH 10 (Figure 5). This pH value, which was used for the determination of the protein solubility among all the hemp seed‐based samples, was based on previous research on protein solubility for hemp seed flour, where a higher protein solubility was found at pH 10 and a lower protein solubility when the samples were treated at pH 4 and 7 (Raikos et al. 2014). In general, a higher pH (10, for example) is associated with the promotion of repulsive forces in proteins, which ultimately prevents protein precipitation, thereby increasing the protein solubility of food samples (Lawal 2004; Moure et al. 2006). The protein solubility values measured for the hemp seed‐based samples at pH 10 (Figure 5) are in accordance with a previous work on protein isolates from hemp seeds, where a higher solubility of the protein was found as the pH of the samples increased to pH 10 (El‐Sohaimy et al. 2022). However, the protein solubility (1.05%–13.73%) of the hemp seed‐based samples analyzed (Figure 5) was lower than that of a work on hemp seed flour (14.80%), which also used a pH of 10 (Raikos et al. 2014). Food matrix effects and the variation in the amount of dietary protein could potentially explain the differences in the results observed for protein solubility. Specifically, the protein content of the hemp seed‐hull flour in this study (Table 1) was 2‐fold lower than that of previously reported hemp seed flour (28%) (Raikos et al. 2014).

Previous publications on Tarom rice bran protein isolate, Shiroodi rice bran protein isolate (Esmaeili et al. 2016), oat flour (Immonen et al. 2021) and barley protein isolate (Yalcin and Celik 2007) were in accordance with our observations found in the hemp seed‐based samples (Figure 5), as they reported similar trends toward an increase in protein solubility as pH increased to 10 (Esmaeili et al. 2016). The protein solubility at pH 10 of the barley protein isolate (18%) (Yalcin and Celik 2007) and oat flour (25%) (Immonen et al. 2021) was similar to that of hemp seed hearts (Figure 5). However, the protein solubility values determined by Esmaeili et al. (2016) were much higher than those found in the hemp seed‐based samples (Figure 5), including the protein‐85‐product, whose protein content (Table 1) was higher than the rice bran samples (Esmaeili et al. 2016). This elevated protein solubility in the rice bran samples may be attributed to the formation of smaller peptide molecules during alkaline extraction (Esmaeili et al. 2016). In addition, in our study (Figure 5), the pH was controlled by using phosphate buffer, while in the work of rice bran isolate samples (Esmaeili et al. 2016), the pH was directly modified using only distilled water. Despite this limitation, hemp protein remains attractive due to its complete amino acid profile, hypoallergenic nature, and environmental sustainability, making it a valuable alternative in solid or semi‐solid food formulations where solubility is less critical.

### 3.8 Lipid Oxidation

**FIGURE 6:**
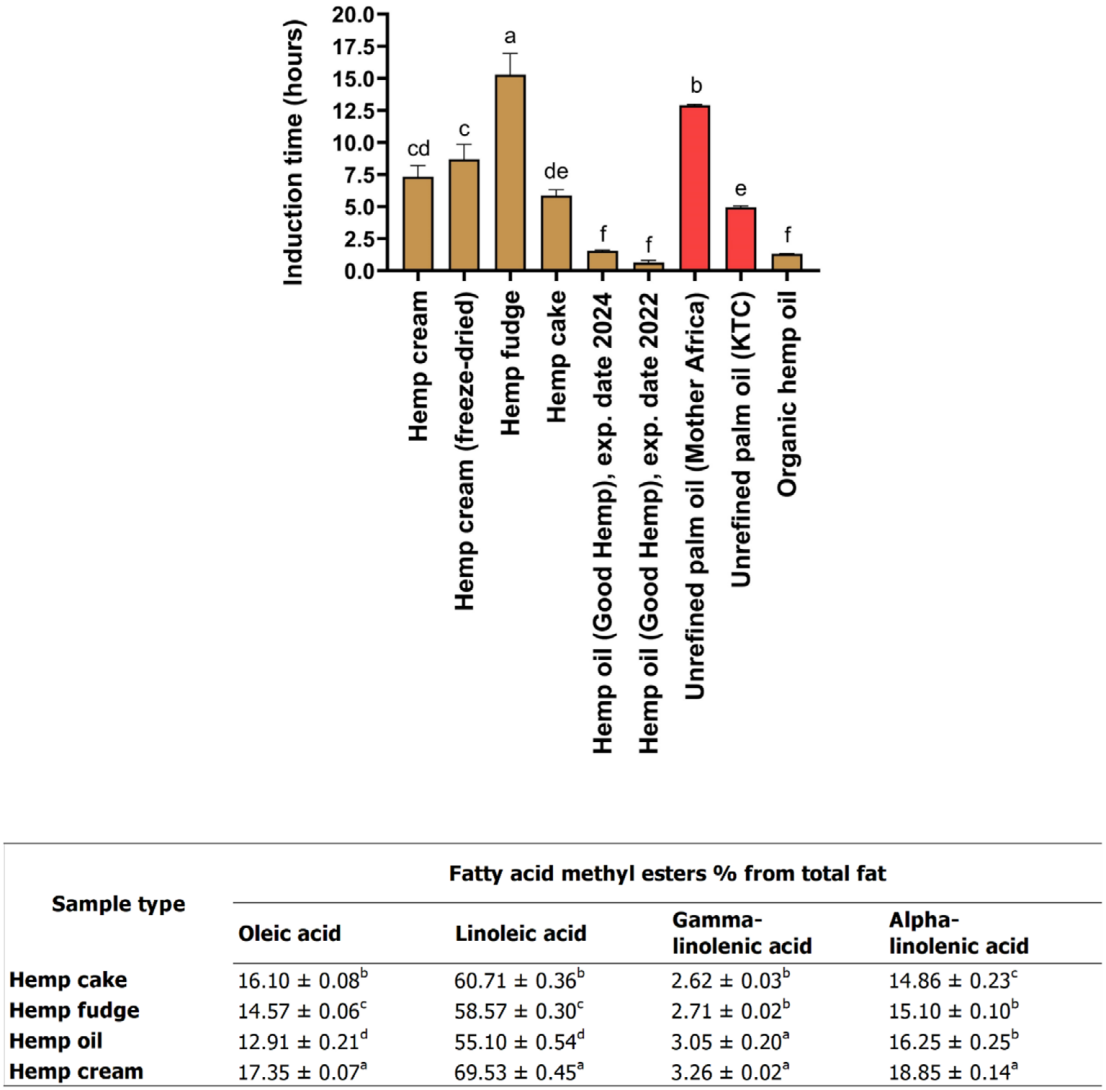
Induction time (hours) of different hemp seed‐based samples, unrefined palm oils, and hemp oils along with the fatty acid methyl esters (%) of distinct types of hemp seed‐based samples, expressed as means (±SD, *n* = 3). Distinct letters above the bars (a–f) indicate statistically significant differences (*p* < 0.05). The fatty acid methyl esters among all the hemp seed‐based samples have been previously published (Ramos‐Sanchez et al. 2025).

The induction time (hours) was used to judge the oxidative stability in organic hemp oil, unrefined palm oils, and several types of hemp seed‐based samples (Figure 6). The induction time in the hemp fudge (15.27 ± 1.65 h) was significantly higher (*p* < 0.05) than that of the rest of the samples and controls (unrefined palm oils from different brands). Previous work has shown that food samples are more likely to oxidize when they contain a higher composition of unsaturated and polyunsaturated fatty acids (Kaseke et al. 2020; Stanković and Radovanović 2012; Symoniuk et al. 2018). In this regard, the increased stability of hemp fudge in reducing lipid oxidation (Figure 6) may be because hemp fudge contained significantly (*p* < 0.05) lower amounts of oleic acid (14.57% ± 0.06%) and linoleic acid (58.57% ± 0.30%) than hemp cake (16.10% ± 0.08% and 60.71% ± 0.36%) and hemp cream (17.35% ± 0.07% and 69.53% ± 0.45%). Moreover, the capacity of hemp fudge to retard lipid oxidation in comparison to that of hemp cream (Figure 6) may be because it contained significantly (*p* < 0.05) lower amounts of gamma‐linolenic acid (2.71% ± 0.02%) and alpha‐linolenic acid (15.10% ± 0.10%). In turn, the potential of hemp fudge to reduce lipid oxidation in comparison to that of hemp oil may be because it contained significantly (*p* < 0.05) lower amounts of gamma‐linolenic acid (2.71% ± 0.02%).

The unrefined palm oil (Mother Africa) was more stable than the unrefined palm oil (KTC), as its induction time (Figure 6) was significantly higher (12.89 ± 0.06 h) than the one found in the KTC brand (*p* < 0.05). This may be attributed to different factors, including differences in the mechanical processes from which the palm oils were extracted from seeds, as well as the period of time these oils were stored (Dia et al. 2005). It has been shown that the time for storage is positively associated with an increase in moisture content. Water in oils is considered a reactant in fat hydrolysis, which can lead to an increase in free fatty acids in oils, thereby resulting in an increase in rancidity (Man and Ali 1992). Therefore, it could be suggested that the differences observed in the induction time (Figure 6) between unrefined palm oils (Mother Africa) and unrefined oil (KTC) may be attributed to differences in moisture content; however, further experiments are necessary to elucidate this.

The induction time between the hemp cream (7.32 ± 0.86 h) and the hemp cream (freeze‐dried, with 8.69 ± 1.16 h) was similar, which means the amount of water was not a factor influencing the oxidation stability results (Figure 6). Hemp cream was more resistant to oxidation than hemp oil (Figure 6), even when hemp cream had a significantly higher profile (*p* < 0.05) of unsaturated and polyunsaturated fatty acids (oleic acid, alpha‐linolenic acid, and linoleic acid). Different types of factors during processing conditions have been reported to influence oxidation stability in food samples, including temperature, pressure, and type of solvent (Pereira et al. 2019). Therefore, the variation in the respective induction times between hemp cream and hemp oil may be attributed to the type of manufacturing process employed to produce these samples. In this regard, the hemp oil is obtained through a cold‐pressed extraction of hemp seeds, while the hemp cream is obtained through a process of milling and pasteurization of the hemp seed hearts.

### 3.9 Potential Industrial Food Uses of Hemp Seed‐Based Samples

The use of synthetic fertilizers and deforestation to meet demand for soya and wheat poses a significant threat to climate change (Jurcuț et al. 2023; Kheiralipour et al. 2024), calling for the development and introduction of food ingredients with a reduced carbon footprint. This study highlights the potential of various types of hemp seed‐based samples to be revalorized by the food industry in relation to their hydration properties, including wettability, solubility, and dispersibility, as these were found to be similar to or even higher than those of the controls (toasted soya flour and wheat flour). Specifically, protein‐75‐product, protein‐85‐product, and hemp cake exhibited the best wettability properties among all the hemp samples and controls (Table 1). The cream solid residue (wet and dried), protein‐75‐product, protein fiber boost, and hemp seed hearts showed to have higher solubility and dispersibility properties compared to the rest of the hemp samples and controls (Figure 4a,b). Therefore, due to their hydration properties, these aforementioned samples contain a lower level of agglomeration, which could potentially be harnessed in food powders intended to produce smoothies, porridges, and bakery products where they could replace soya and wheat.

The protein solubility represents a critical parameter for the revalorization of the hemp seed‐based samples. For instance, it plays an important role in determining emulsifying and foaming capacities in food products (El‐Sohaimy et al. 2022). Moreover, protein solubility plays an important role in regulating the physicochemical characteristics, processing, sensory qualities, shelf life, and nutritional composition of foods (Grossmann and McClements 2023). Herein, the hemp seed‐based samples exhibited increased protein solubility as pH rose. Notably, the hemp seed hearts exhibited the highest protein solubility, with values similar to those of common ingredients such as barley protein isolate and oat flour. These results suggest a strong potential of the hemp seed‐based samples, mainly the hemp seed hearts, protein fiber boost, and expellers, to be revalorized within the food industry for use in emulsified and foamed food products, where enhanced sensory properties are desirable.

WHC refers to the ability of food matrices to absorb and retain water even during different food processing processes, including slicing or cooking (Dekkers et al. 2018; Warner 2017). For instance, WHC plays a crucial role in the production of emulsion‐type sausages, as it prevents the denaturalization of protein and fat melting (Kyriakopoulou et al. 2021). Moreover, a correct balance between protein and water enables water to remain in the voids of the protein network after heating, resulting in final products with desired texture and juiciness characteristics (Kyriakopoulou et al. 2021). In turn, WHC is important in the production of food products where food ingredients are used as binder agents (Torres Vargas et al. 2018). Previous work has reported the use of wheat and soya flours as binders in the processing of beef sausages (Babatunde et al. 2013; Elbakheet et al. 2018). Apart from that, food ingredients with high OHC are desirable to improve sensory characteristics of different food products, including meat analogues and sausages (Kaleda et al. 2020; Tarahi et al. 2024). Herein, the capacity of protein‐75‐product, protein‐85‐product, and protein‐46‐product to retain water was better than that of wheat flour (Figure 4c). Besides, the OHC of all the hemp samples analyzed was comparable to that of toasted soya flour and wheat flour (Figure 4d). Therefore, these hemp samples may be revalorized by the meat food industry as alternative binder options to replace the use of wheat and soya flours.

Palm fat is high in saturated fats, particularly palmitic acid, which has been linked to increased LDL cholesterol levels and a higher risk of cardiovascular disease when consumed in excess (Mensink et al. 2003). Additionally, palm oil production is a major driver of tropical deforestation, habitat loss, and significant greenhouse gas emissions (Vijay et al. 2016). Therefore, new eco‐friendly substitutes for palm fat are needed to reduce carbon emissions. Herein, the lipid stability of hemp fudge and hemp cream (Figure 6) was much higher than that of unrefined palm oil (KTC); therefore, these hemp samples could be explored by the food industry and used as innovative and healthier substitutes for palm fat. Moreover, hemp cake was more stable against lipid oxidation than unrefined palm oil (KTC), which may be harnessed by the food industry to produce bakery products where thermal temperatures are usually high.

## 4 Conclusions

The findings of this study underscore the strong potential of hemp seed‐based ingredients for revalorization and practical integration into food industry applications, particularly as functional alternatives to conventional ingredients such as toasted soya flour, wheat flour, and palm fat. Notably, the comparable loose and tapped bulk densities of most hemp samples to those of soya and wheat flours suggest that substituting these ingredients with hemp‐based alternatives is unlikely to incur significant increases in transportation costs, an important consideration for commercial scalability.

Several hemp‐derived products, including Protein‐75, Protein‐85, hemp cake, cream solid residue (both wet and dried), Protein Fiber Boost, and hemp seed hearts, demonstrated superior hydration properties and reduced agglomeration. These functional traits are especially advantageous for incorporation into moisture‐rich formulations such as smoothies, porridges, and baked goods, where rapid dispersion and consistent texture are critical.

Moreover, the enhanced water and fat retention capacities observed in Protein‐75, Protein‐85, and Protein‐46 highlight their suitability as natural binders in the development of meat analogues and plant‐based sausages. These properties contribute to improved product structure, mouthfeel, and shelf stability, key factors in consumer acceptance and market competitiveness.

The high protein solubility exhibited by hemp seed hearts, Protein Fiber Boost, and expellers further reinforces their value in food reformulation. Protein solubility is a pivotal attribute for emulsified and foamed products, as it directly influences the stability of dispersed phases. This positions these ingredients as promising candidates for use in plant‐based dairy alternatives, sauces, and aerated desserts.

Importantly, hemp protein offers a significant advantage over soya‐based ingredients in terms of allergenicity. As a non‐allergenic protein source, hemp is suitable for a broader consumer base, including individuals with soy allergies, thereby enhancing product inclusivity and market reach. Additionally, hemp cultivation is more environmentally sustainable, requiring fewer inputs such as water and pesticides, and contributing to soil health through phytoremediation, making it a compelling choice for eco‐conscious food innovation.

Key physical properties such as wettability, flowability, bulk density, and WHC were influenced by the protein and fat content, as well as the particle size of the samples. While higher levels of unsaturated fatty acids can predispose products to lipid oxidation, certain hemp‐based ingredients, specifically Hemp Fudge, Hemp Cream, and Hemp Cake, exhibited superior lipid stability compared to commercial palm oil (KTC). This finding supports the use of Hemp Fudge and Hemp Cream as viable replacements for palm fat in spreads, fillings, and confectionery, while Hemp Cake emerges as a particularly suitable ingredient for bakery applications such as muffins, cookies, and high‐fiber bread.

In summary, the diverse functional properties, nutritional benefits, hypoallergenic profile, and sustainability of hemp seed‐based ingredients make them highly relevant for future food reformulation. Their versatility supports the development of innovative, clean‐label, and plant‐based products that align with both industry needs and evolving consumer preferences.

## Author Contributions

**Ricardo Ramos‐Sanchez:** conceptualization (equal), data curation (lead), formal analysis (lead), methodology (equal), writing – original draft (equal), writing – review and editing (equal). **Nicholas J. Hayward:** data curation (equal), formal analysis (equal), methodology (equal). **Wendy R. Russell:** conceptualization (equal), investigation (equal), methodology (equal), supervision (equal), writing – review and editing (equal). **Sylvia H. Duncan:** conceptualization (equal), funding acquisition (equal), investigation (equal), methodology (equal), writing – original draft (equal), writing – review and editing (equal). **Madalina Neacsu:** conceptualization (lead), funding acquisition (lead), investigation (equal), methodology (equal), project administration (lead), supervision (lead), writing – original draft (equal), writing – review and editing (equal).

## Ethics Statement

The authors have nothing to report.

## Consent

The authors have nothing to report.

## Conflicts of Interest

The authors declare no conflicts of interest.

## Supporting information


**Figure S1:** SEM images of different hemp seed‐based samples and controls. (a) hemp protein fiber boost; (b) protein‐75‐product; (c) protein‐85‐product; (d) protein‐46‐product; (e) hemp seed‐hull flour; (f) hemp seed hearts; (g) expellers; (h) seeds; (i) hemp cake; (j) cream solid residue (wet); (k) cream solid residue (dried); (l) toasted soya flour; (m) wheat flour.

## Data Availability

The document contains the necessary data.
