# Supplementary material for: Evaluation of Protein Solubility, Lipid Oxidative Stability and Physical Properties of Hemp Seed‐Based Foods and By‐Products
Source: Food Sci Nutr. 2025 Sep 10;13(9):e70954. doi: 10.1002/fsn3.70954 (PMC12421313; doi:10.1002/fsn3.70954)
Supplement: Supplementary file 1 — Figure S1: SEM images of different hemp seed‐based samples and controls. (a) hemp protein fiber boost; (b) protein‐75‐product; (c) protein‐85‐product; (d) protein‐46‐product; (e) hemp seed‐hull flour; (f) hemp seed hearts; (g) expellers; (h) seeds; (i) hemp cake; (j) cream solid residue (wet); (k) cream solid residue (dried); (l) toasted soya flour; (m) wheat flour. [file FSN3-13-e70954-s001.docx]

**a) Protein fibre boost**


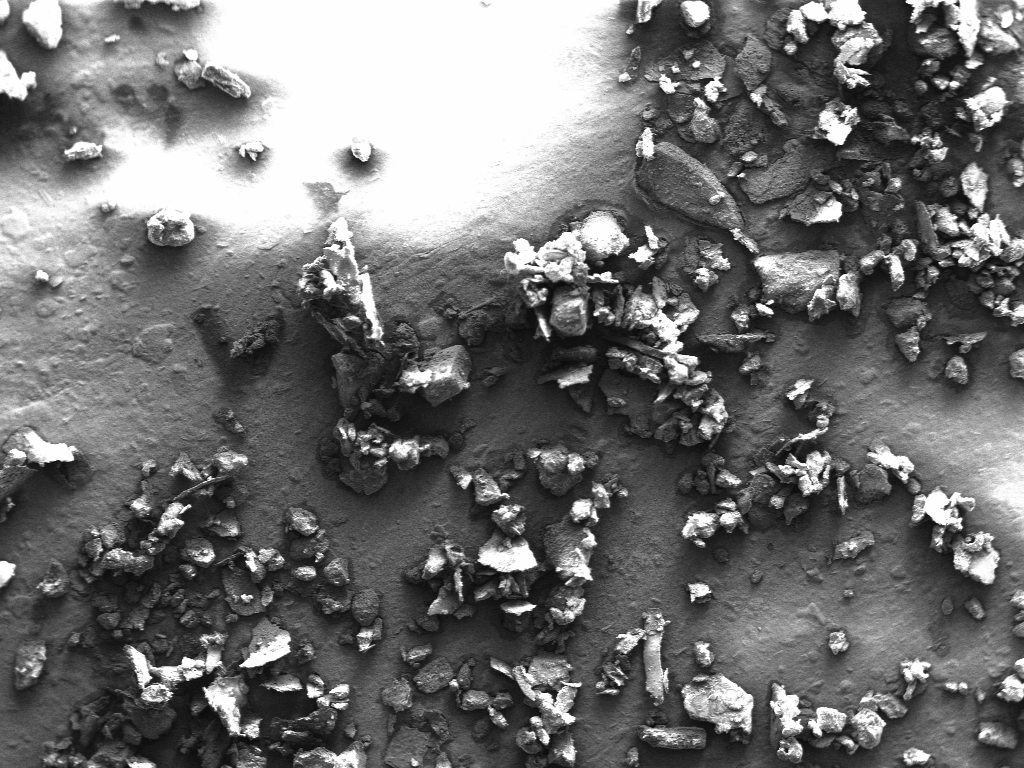


**b) protein-75-product**


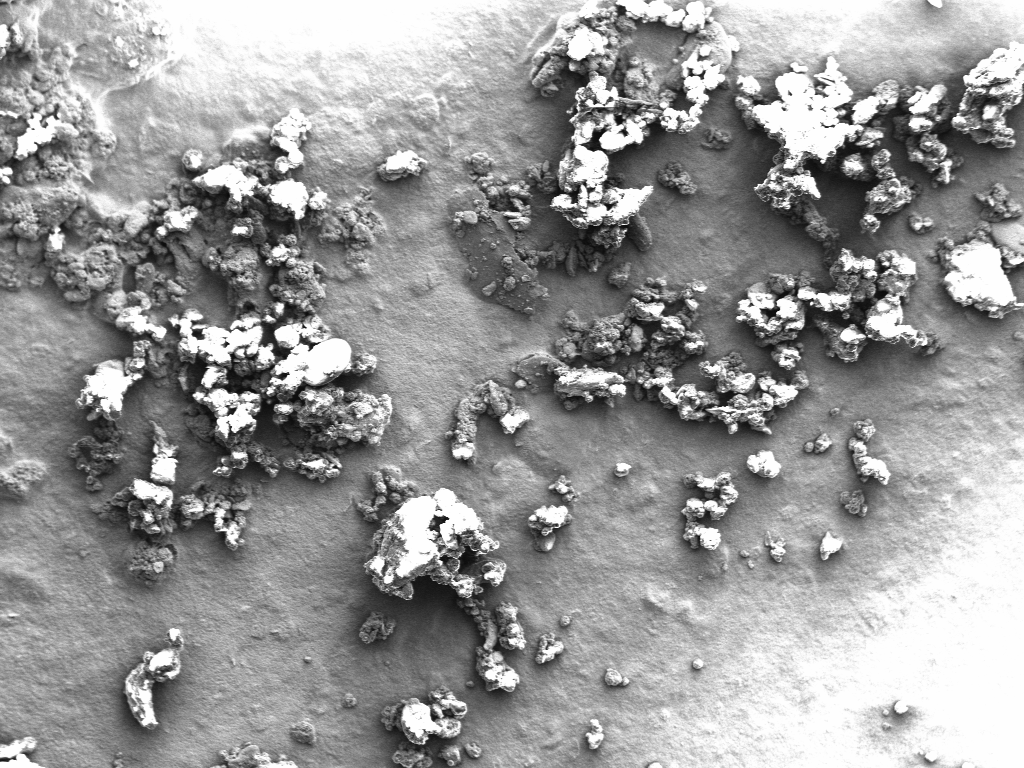

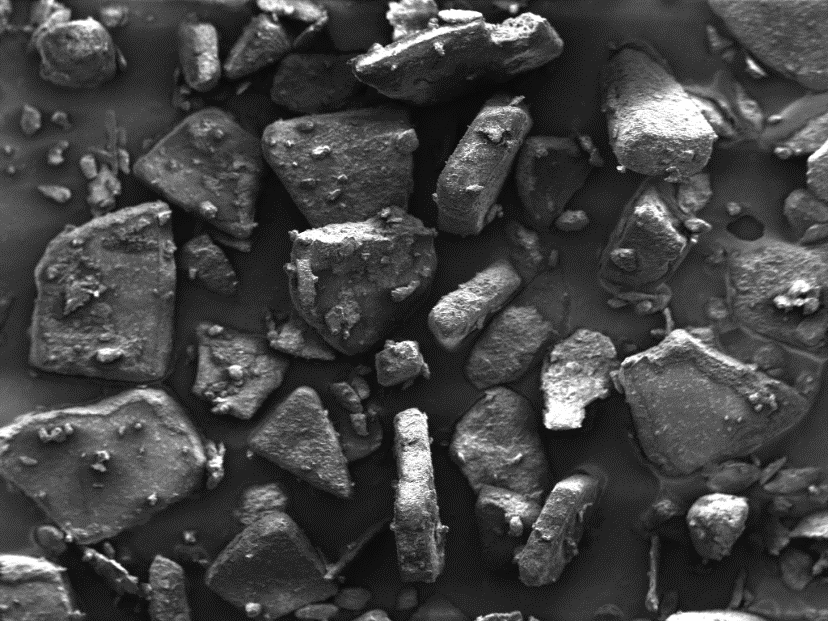


**e) hemp seed-hull flour**

**d) protein-46-product**


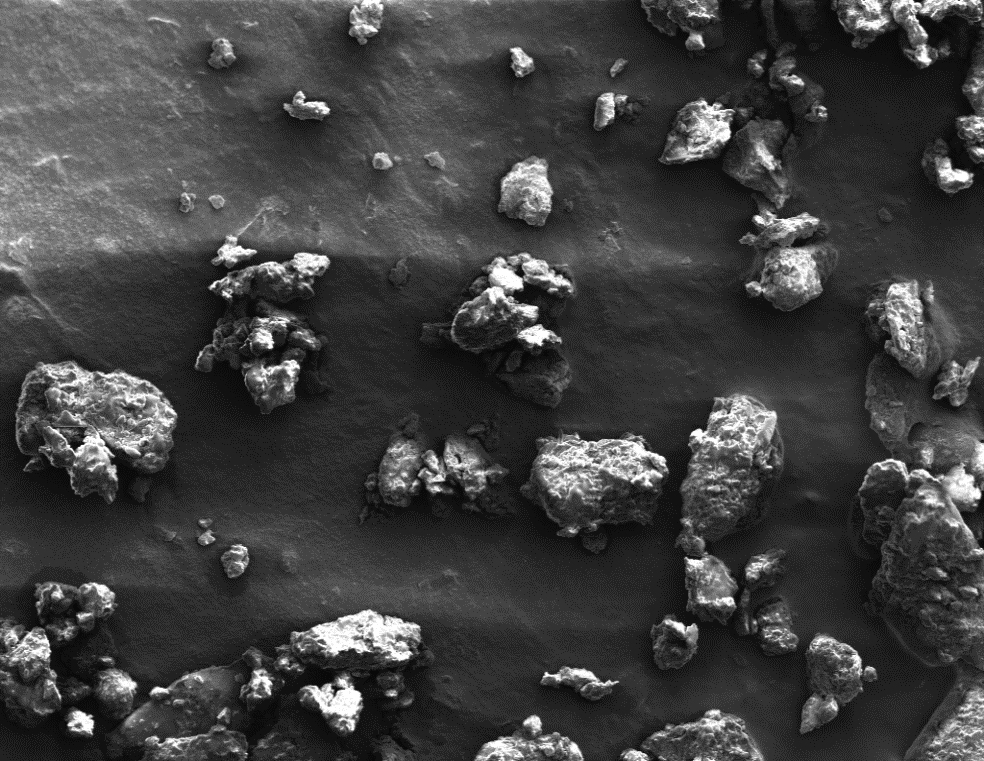


**c) protein-85-product**


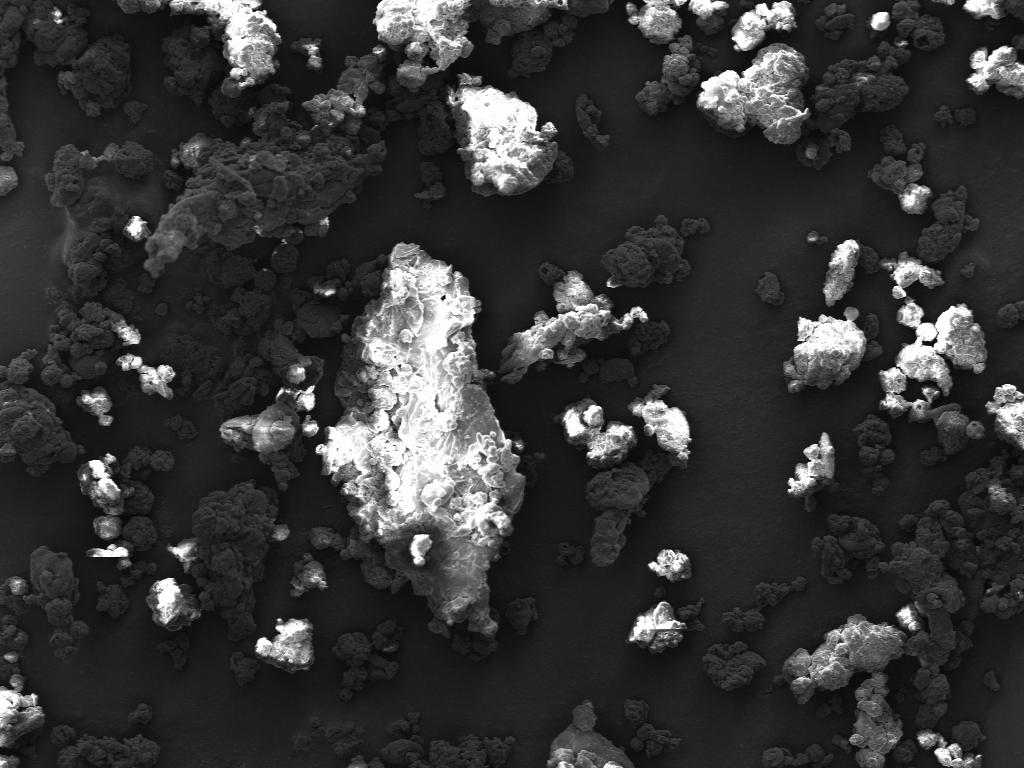


**f) hemp seed hearts**


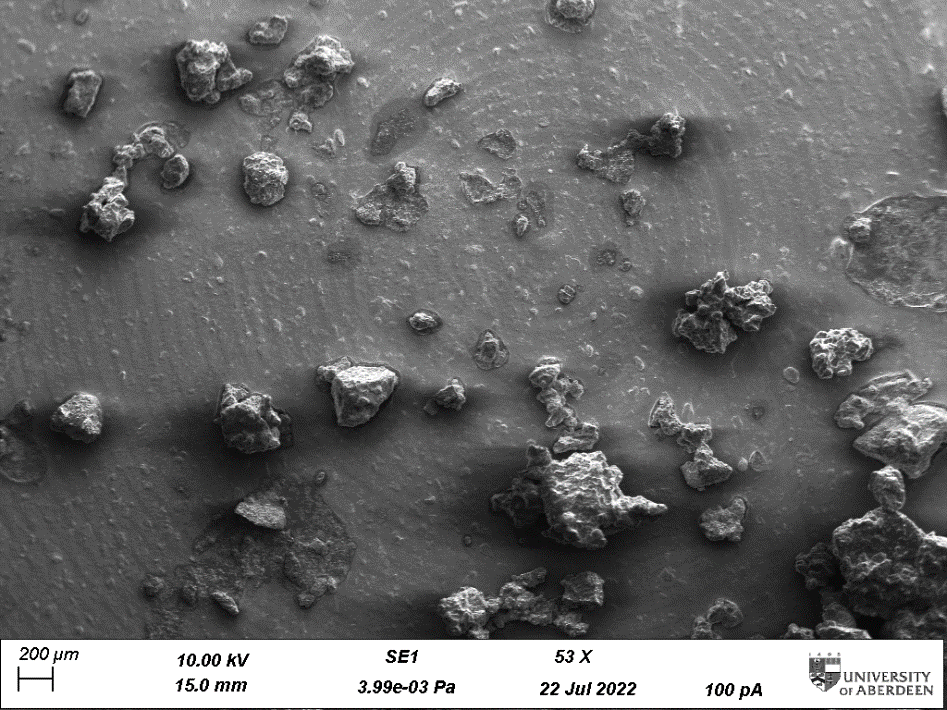


**g) expellers**


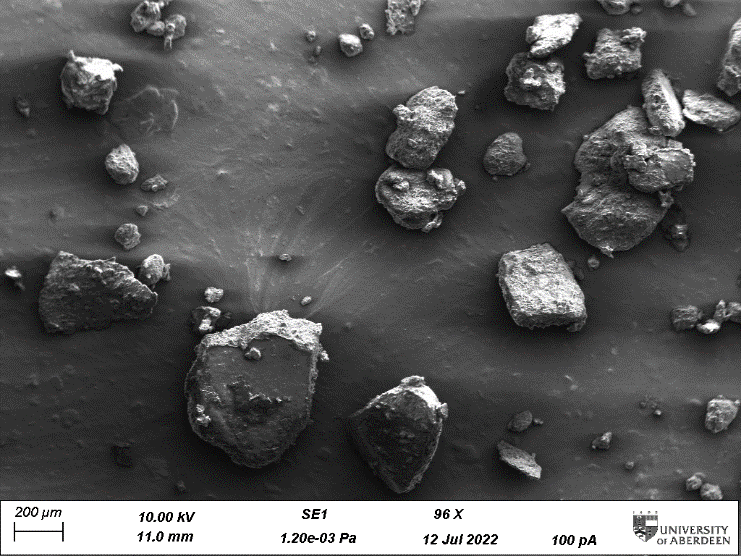


**h) seeds**


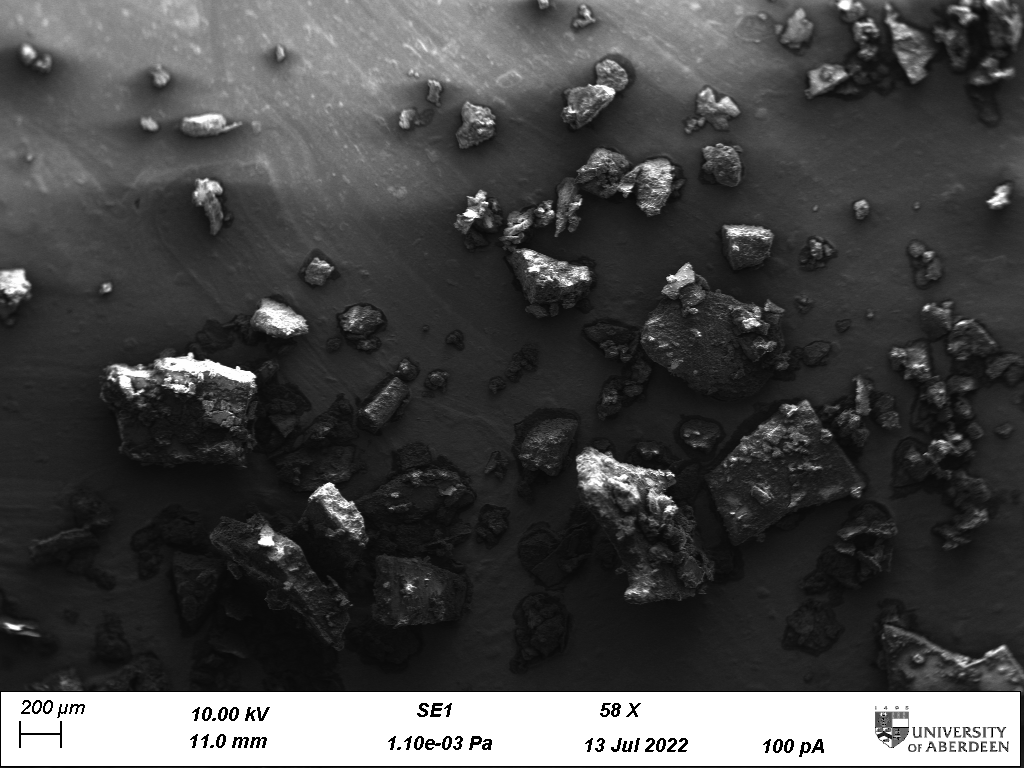


**k) cream solid residue (dried)**


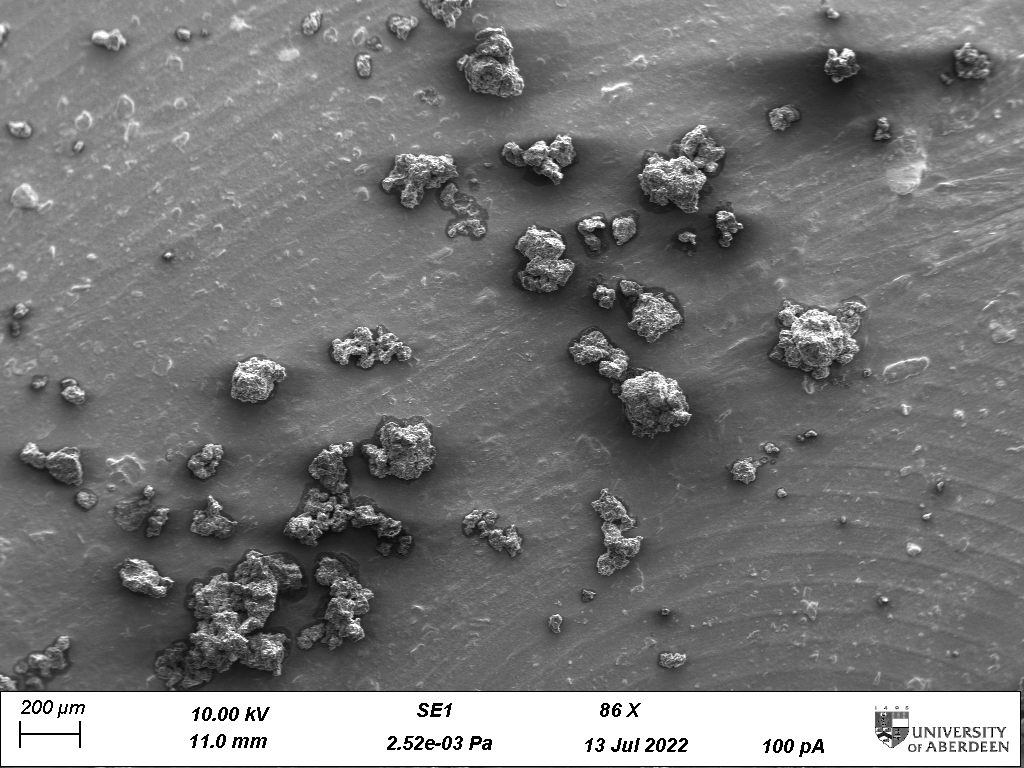

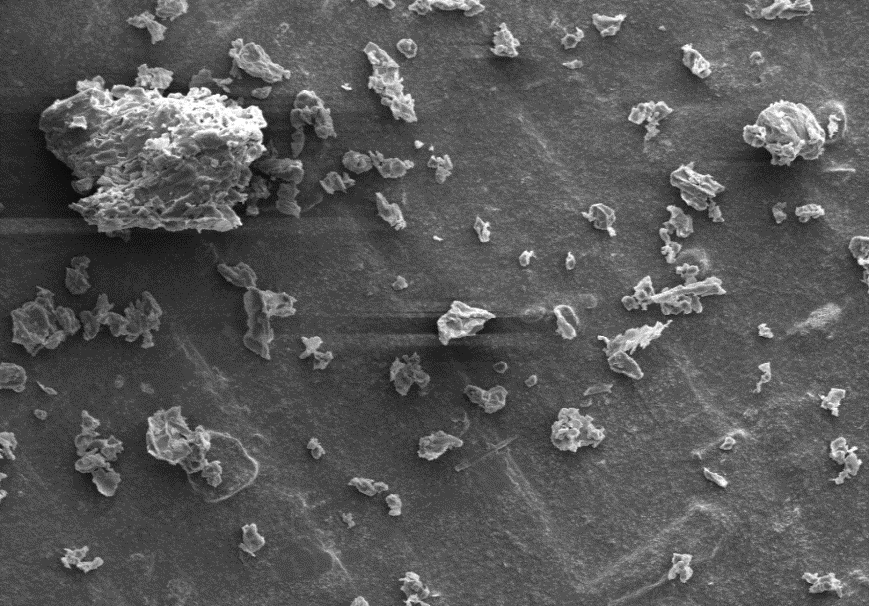


**l) soya flour toasted**


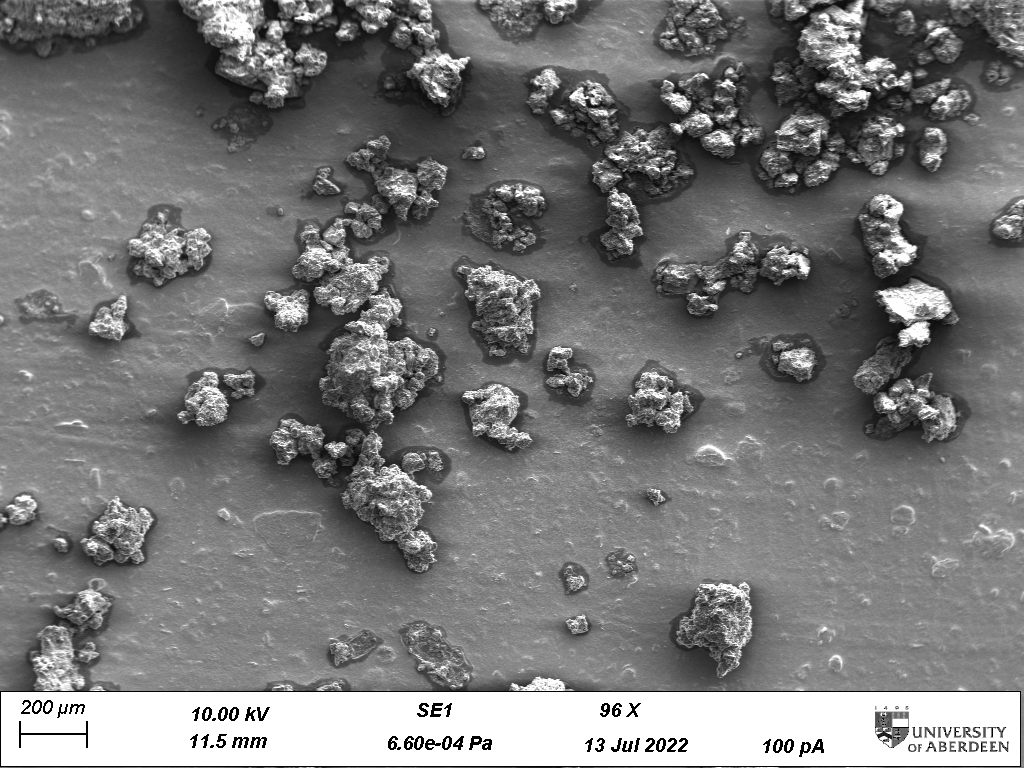


**j) cream solid residue (wet)**

**i) hemp cake**


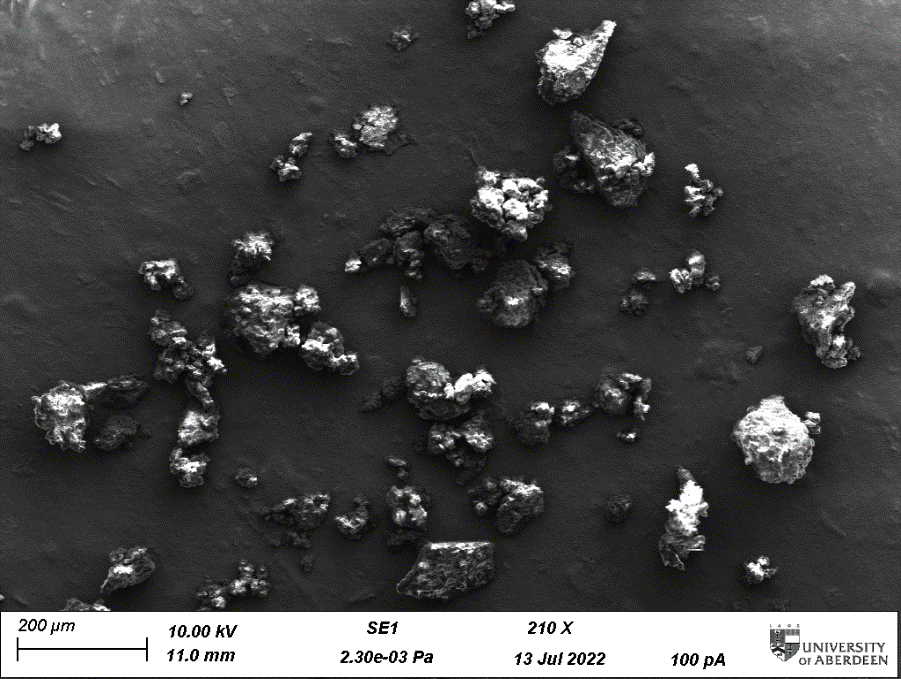


**m) wheat flour**


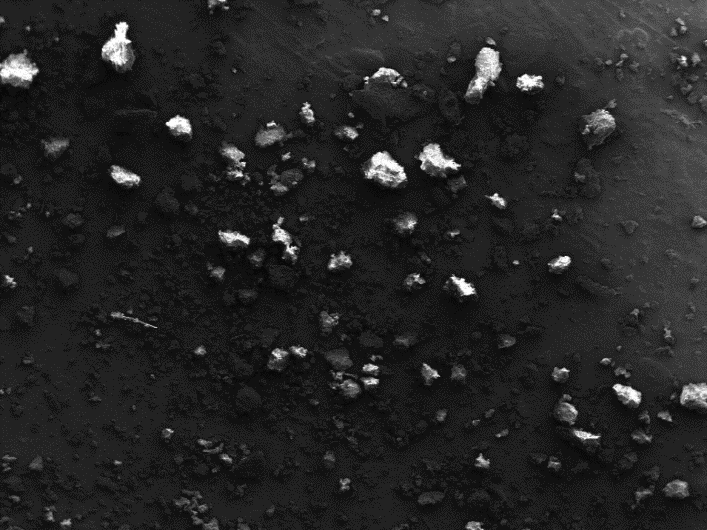


Figure S1. SEM images of different hemp seed-based samples and controls. a) hemp protein fibre boost; b) protein-75-product; c) protein-85-product; d) protein-46-product; e) hemp seed-hull flour; f) hemp seed hearts; g) expellers; h) seeds; i) hemp cake; j) cream solid residue (wet); k) cream solid residue (dried); l) soya flour toasted; m) wheat flour.
